# Removal of Copper Corrosion Products by Using Green Deep Eutectic Solvent and Bio-Derivative Cellulose Membrane

**DOI:** 10.3390/polym14112284

**Published:** 2022-06-04

**Authors:** Akiko Tsurumaki, Cristina Chiarucci, Shraddha Khaire, Chiara Dal Bosco, Alessandra Gentili, Maria Assunta Navarra

**Affiliations:** 1 Department of Chemistry, Sapienza University of Rome, Piazzale Aldo Moro 5, 00185 Rome, Italy; chiaruccicristina@gmail.com (C.C.); skhaire2095@gmail.com (S.K.); chiara.dalbosco@uniroma1.it (C.D.B.); alessandra.gentili@uniroma1.it (A.G.); 2 Centro di Ricerca Hydro-Eco, Department of Basic and Applied Sciences for Engineering (SBAI), Sapienza University of Rome, Via Antonio Scarpa 16, 00161 Rome, Italy

**Keywords:** corrosion removal, electrochemical corrosion, copper artifacts, deep eutectic solvent (DES)

## Abstract

A safe and environmentally friendly material for corrosion removal from metals is proposed in this article. Electrochemically corroded copper was selected as a target material, and a deep eutectic solvent (DES) composed of choline chloride and ascorbic acid, in a molar ratio of 2:1, was developed to this end. Aqueous solutions of the DES with a concentration above 70 wt% were found to be effective in the dissolution of patina and less aggressive towards other materials such as CaCO_3,_ which is the main component of limestone. These concentrated DES solutions were integrated with either cotton swabs or cellulose-based membranes and used for the cleaning of electrochemically corroded copper. The membrane containing 80 wt% DES aqueous solution exhibited the most desirable cleaning ability in terms of speed and area selectivity. X-ray diffraction analysis of the corroded copper before and after the application of the membrane was performed to demonstrate the successful corrosion removal.

## 1. Introduction

With technological and scientific progress, there is a strong expectation for greener and safer procedures for the removal of corrosion products such as from ancient metal artifacts. Copper was one of the first metals to be used by humans; copper-based artifacts have been the most frequently found objects in archaeological sites throughout history [1] and are generally found with a layer of naturally developed patina on their surfaces. Without any specific treatment, corrosion continuously spreads over the objects and destroys their fine structures. During corrosion removal, the level of cleaning, i.e., the layer required to be removed, must be considered and controlled. One of the most well-known issues with the preservation of copper-based materials is the removal of a reddish-brown cuprite layer because this layer buries the surface texture of the object but, at the same time, it works as a protective layer against further damages [2,3]. Therefore, the level of cleaning in terms of the selectivity of both area and depth needs to be carefully controlled depending on demands and circumstances. This should be considered not only in the field of heritage conservation but also in the industrial field.

The cleaning of copper has been carried out via both physical and chemical procedures. When the corrosion layer is thick, the physical cleaning using a hard and stiff nylon brush is generally carried out prior to the chemical cleaning. For the chemical cleaning, some reagents, such as ethylenediaminetetraacetic acid (EDTA) and sodium tripolyphosphate (STPP) are used to remove green patinas [4,5,6]. EDTA is reported to have a strong dissolution ability of patina through the formation of a complex with Cu^2+^, and compared to this, STPP is known to be less effective in the dissolution of malachite and cuprite but less harmful to the base metal [6]. Although these chemicals effectively remove copper corrosion, they have a detrimental impact on the environment. For instance, EDTA is one of the anthropogenic compounds found in inland European water [7]. Also, the use of harmful chemicals should be avoided during copper cleaning for both objects and conservators. Therefore, safer and greener cleaning agents and some bio-derived acids, such as citric acids and tartaric acids, have been taken into consideration [8]. However, their acidity needs to be carefully controlled in order to protect the metal surface from etching, and this has been achieved by combining acids with a buffer solution, such as ammonium hydroxide [9].

With the aim of developing greener and safer chemical cleaning methods for copper corrosion, composite materials merely based on bio-derived components, specifically deep eutectic solvents (DESs), sodium carboxymethylcellulose (NaCMC), and 2-hydroxyethyl cellulose (HEC), are proposed in this study. DESs are systems formed from a eutectic mixture of Lewis or Brønsted acids and bases [10], which are recently acknowledged to be environment-friendly solvents and used for a variety of applications, from the extraction of bio-derived materials to energy storage systems [11,12,13]. The starting components of DES are solid, but their proper combination produces a room temperature liquid with a low volatility through the formation of strong hydrogen bonds. In general, Lewis acids such as urea serve a function as a hydrogen bond donor (HBD) and form a complex with the halide anion of a quaternary ammonium salt added as a hydrogen bond acceptor (HBA). This complexation affects the charge delocalization and decreases in the melting point of the mixture [10]. It is reported that the nanostructure of DES maintained by hydrogen bonds is preserved even at a remarkably high level of water content (ca. 42 wt% H_2_O) [14]. DESs composed of solely bio-derived materials can be produced, e.g., choline chloride (ChCl) and urea [15], which is the most widely studied eutectic mixture. In the industrial field, DESs are currently used for metal processing [10,16,17], such as metal deposition, metal dissolution, or rust removal. On the other hand, in the field of cultural heritage conservation, DESs have only been applied as temporary consolidants [18] and cleaning agents for organic varnishes [19]. To the best of our knowledge, their use in the removal of metal corrosion products represents a new frontier in this research field. As the conservation of cultural heritage often involves manual cleaning of materials on site, the solvents used for this purpose must be environmentally friendly and safe. These are the characteristics in which DESs excel.

In this study, a DES was prepared by mixing ChCl as a HBA and ascorbic acid as a HBD in a molar ratio of 2:1. Once formed, DES was used for the removal of malachite-based corrosion products from copper for the first time. A gentle cleaning protocol was developed by integrating DES with a membrane based on NaCMC and HEC, thus improving the selectivity of the cleaning system towards the areas that require specific treatment.

## 2. Materials and Methods

A series of DES aq. soln. was prepared as follows. Choline chloride (ChCl) and L-ascorbic acid (AA) were purchased from Merck KGaA and used without any purification. Both reagents were dried at 60 °C for a week and mixed to reach the concentration of ChCl:AA = 2:1 mol/mol under ambient conditions. The mixture was then heated at 80 °C for 30 min to form the DES. The concentration of water in the DES was 4 wt%, which was determined by means of isothermal thermogravimetric analysis at 110 °C. Taking the intrinsic water concentration into consideration, the following DES aqueous solutions were prepared and named as DES10, 30, 50, 70, 80, and 90, with the numbers denoting the resulting concentration of DES.

Membranes based on NaCMC and HEC were prepared using citric acid (CA) as a crosslinking agent as reported in the literature [20]. The NaCMC with Mw ~700,000, HEC with average Mw ~250,000, and CA were purchased from Merck KGaA and used as received. Then, 98 mL of distilled water and 115 mg of CA, which is equivalent to 5.75 wt% per combined mass of polymers, were mixed in a weighing bottle with 8 cm diameter. After complete dissolution of CA, 1.5 g of NaCMC and 0.5 g of HEC were added and stirred slowly overnight at room temperature. After removing the stirring magnet, the weighing bottle was kept at 40 °C for 5 days to remove water. Then, it was heated at 80 °C for 24 h for crosslink formations. The average thickness of resulting membrane was 0.125 mm.

To prepare electrochemical corrosion on copper, copper sheets (0.127 cm thickness, 3 cm width × 6 cm length) were rinsed with 0.01 M H_2_SO_4_. Then, they were immersed in the trianionic solution which contained 0.01 M Na_2_SO_4_, 2.8 × 10^−2^ M NaCl, and 16.1 × 10^−2^ M NaHCO_3_ [21,22]. Using platinum wire as a counter electrode and Ag/AgCl as a reference electrode, an anodic potential of 0.150 V (vs. Ag/AgCl) was applied to the copper sheets for 2 h, followed by a potential of 0.380 V (vs. Ag/AgCl) for 96 h. The corroded copper was rinsed with acetone and dried at 60 °C for 1 day.

Dissolution test of copper corrosion products and calcium carbonate in the series of DES aq. was carried out as follows. The patina was scratched off from the electrochemically corroded copper sheets and manually grinded using an agate mortar. Calcium carbonate (≥99.0%) was purchased from Merck KGaA. To 300 mg of DES aq., 3.0 mg of either copper corrosion powder or calcium carbonate was added. The samples were kept at room temperature without stirring, and their solubility was visually evaluated every hour for the first 5 h and every day for the next 6 days.

Swelling ratio of cellulose-based membranes in DES aq. was calculated in the following manner. The membranes were cut into 1 cm diameter circles. These disks (having a weight between 10.1 and 13.1 mg) were immersed in 500 mg DES aq. (specifically, DES70, DES80, and DES90). The weight of membranes was evaluated every hour for the first 5 h and every day for at least next 7 days. The swelling ratio was calculated according to the following equation:

$$\mathrm{Swelling}\;\mathrm{ratio}\;\left(\%\right)=\frac{w_{s}-w_{d}}{w_{d}}*100$$

in which, *w*_s_ and *w_d_* are the weight of swollen and dried membrane, respectively.

Cleaning of electrochemically corroded copper sheets was performed in two ways. First, cleaning was carried out simply with swabs. Using the cotton balls (diameter ~5 mm and weight ~15 mg) that soaked up around 60 mg of the DES solution, the corroded copper surface was wiped 50 times. The samples were then rinsed with acetone and dried at 80 °C for 1 min. These cleaning steps were repeated 5 times using a different cotton ball every time. Second, cleaning was conducted by using the cellulose-based membranes, which were immersed for 1 day in the case of DES70 and DES80 and for 1 week in the case of DES90. The swollen membranes were placed on the corroded copper sheets, which were then covered with a glass plate to avoid the evaporation of water. The membranes were removed from the copper sheets every 1 h in the first 6 h to evaluate cleaning efficiency but were placed on them again for 1 day in the case of DES70 and DES80, and for 4 days in the case of DES90, to finish cleaning. When the removal of corrosion was confirmed, the copper samples were rinsed with acetone and dried at 80 °C for 1 min before taking a photo.

X-ray diffraction analysis of the corroded copper sheets before and after cleaning was performed using a Rigaku d-max Ultima + diffractometer equipped with a CuKα source. The goniometer speed was 1°/min. In the case of cleaned copper, the part that was not covered with the cellulose membrane was cut off to analyze solely the cleaned area.

## 3. Results

### 3.1. Screening of DES Concentration

Dissolution tests of patina powder and calcium carbonate were carried out with the aim of a quick screening of the DES concentration in its aqueous solution. Table 1 summarizes the time required for the complete dissolution of these two materials. The photos taken during the course of evaluation are summarized in the Supporting Information (Supplementary Material Figures S1 and S2). The fast and complete dissolution of patina powders was observed in DES30, DES50, and DES70. This dissolution was accompanied by a formation of bubbles (see the first line of Supplementary Material Figure S1), attributed to CO_2_ [23]. In the most diluted solution, i.e., DES10, complete dissolution was not achieved. The concentrated solutions, such as DES80 and DES90, took a longer time for complete dissolution, which is most likely due to their high viscosity. By keeping the solubilized samples at room temperature, blue or greenish-blue precipitations were formed in the cases of DES10, DES30, and DES50 (Supplementary Material Figure S1). As reported in the literature [24], it was expected that copper (II) ions formed a complex with ChCl and/or AA. The DES70 sample became slightly yellow but precipitation was not formed, and the DES80 and DES90 samples were stable without any changes in their color.

With respect to the solubility of CaCO_3_, complete dissolution was confirmed when a concentration of the DES was between 30 wt% and 70 wt%. Similar to the dissolution of patina, gas was formed during the dissolution (Supplementary Material Figure S2). In contrast to these three samples, the dissolution of calcium carbonate in DES10, DES80, and DES90 was incomplete even after 1 week. It can be concluded that DES80 and DES90 can dissolve patina powders, but they are less harmful to calcium carbonate, which suggests that their dissolution ability is more selective and suitable for the removal of copper corrosions, especially from artifacts. Taking these results into account, a focus was placed on the DES aq. with higher concentrations, specifically DES70, DES80, and DES90, for the following experiments.

### 3.2. Swelling Test of Cellulose-Based Membranes in DES Aq.

**Figure 1:**
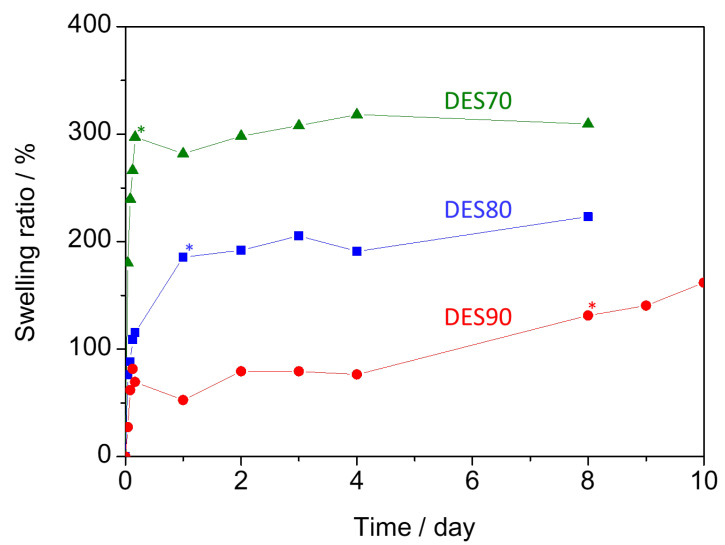
Swelling ratio of cellulose-based membrane in DES70 (triangle), DES80 (square), and DES90 (circle).

Figure 1 summarizes the swelling ratio of cellulose-based membrane immersed in DES70, DES80, and DES90. In their dried state, CMC/HEC membranes were rigid and brittle, which became a gel form by immersing them in the DES aq. The asterisks in the figure designate the time at which the transition of the membrane from solid to gel occurred. The same evaluation was carried out using water as a reference. In this case, the swelling ratio reached at most 1645% after 3 h, and the membrane was broken afterward. The quick disintegration of the membrane suggests its crosslinking density was not high. When the DES aq. solutions were used, the disintegration was suppressed. In the case of DES70, the membrane rapidly absorbed the solution in the first 4 h, and its swelling ratio reached 300%, which was almost constant during the course of the assessment. The membranes in DES80 and DES90 became gels after 1 day and 8 days, respectively. The membrane in DES90 took a much longer time to become swollen than those in DES70 and DES80, and its weight continuously increased, even after the gel formation.

### 3.3. Cleaning of Corroded Copper with DES Aq.

**Figure 2:**
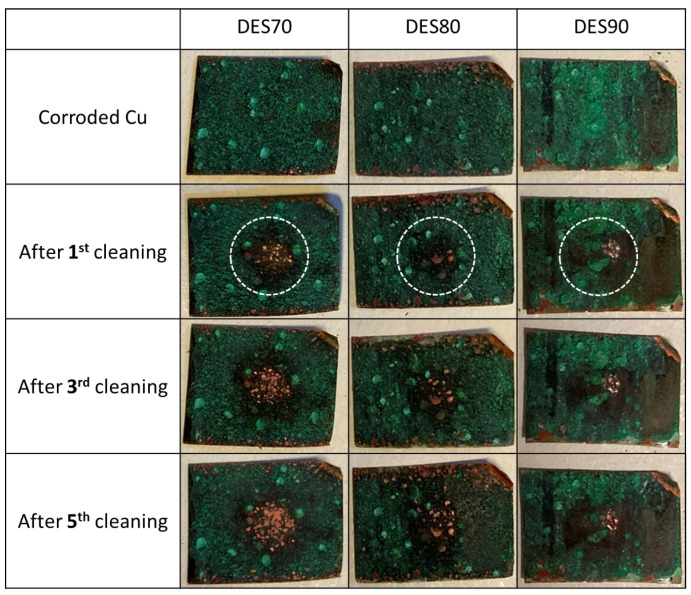
Photos of corroded copper sheets before cleaning, as well as after 1st, 3rd, and 5th cycle of cleaning. Circles with broken white lines designate the area cleaned with cotton swabs containing DES aq.

Corrosion removal from electrochemically corroded copper was undertaken in two ways. First, corroded copper sheets were cleaned by cotton swabs containing either DES70, DES80, or DES90. Second, the abovementioned swollen membranes were used. Figure 2 shows the results of cotton swab cleaning. All solutions are confirmed to be effective for the removal of corrosion even after the first cycle of cleaning. The efficiency of cleaning was better for DES70 compared to DES80 and DES90, which is consistent with the results of patina dissolution. By repeating the cleaning procedure five times with DES70, a surface of metallic copper was uncovered. When DES80 was used, a patina layer including malachite was removed, but a major part of the cuprite layer remained even after cleaning five times (refer to Section 3.4 for corrosion species).

**Figure 3:**
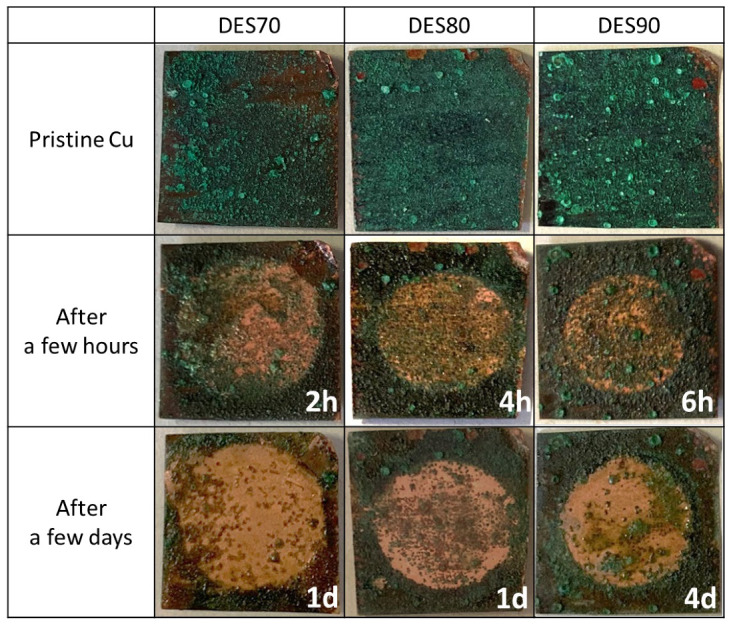
Photos of corroded copper sheets before and after cleaning with cellulose membranes (diameter was 1 cm) containing DES70, DES80, and DES90.

Cleaning using cellulose membranes, which swelled in the DES aq., was also attempted (Figure 3). The membranes absorbing 32.7 mg, 20.8 mg, and 10.0 mg of DES70, DES80, and DES90, respectively, were placed on the corroded copper. In the first 6 h, the membranes were removed every 1 h to check the cleaning efficiency. Through the reaction of green patina and the DES in the membranes, the green malachite layer was successfully removed from the copper sheet by removing the membrane. The time required to achieve sufficient removal was 2 h, 4 h, and 6 h using the membrane that swelled in DES70, DES80, and DES90, respectively. When DES70 was used as a cleaning solvent, the color of patina that was not covered by the membrane was also altered. This is due to a bleed out of the DES aq. from the polymer matrix as a consequence of its relatively low viscosity. Therefore, area selectivity in the cleaning was better in the case of the membranes with DES80 and DES90. After keeping the membrane on the copper for 1–4 days, metallic copper was exposed in all cases.

### 3.4. XRD Analysis of Corroded Copper before and after Cleaning

**Figure 4:**
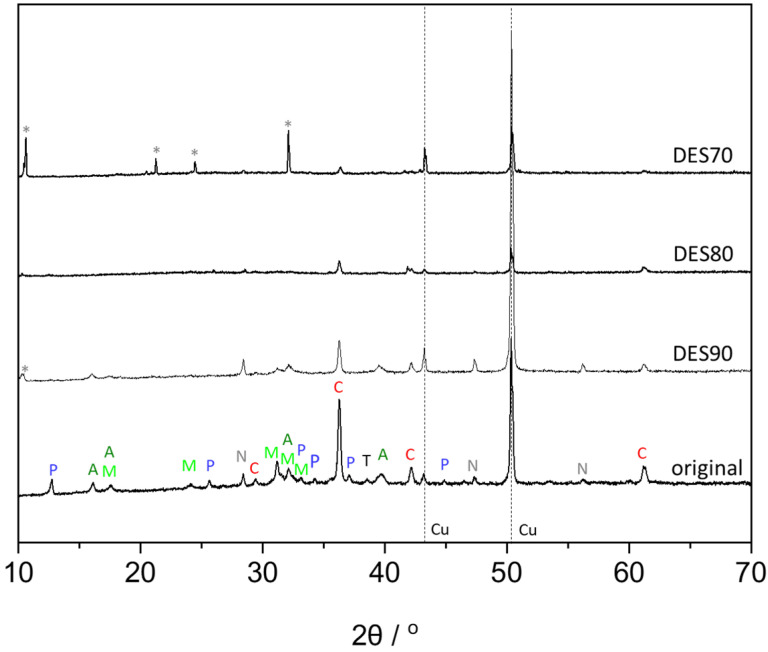
XRD spectra of electrochemically corroded copper sheets before and after corrosion removal using DES aq. absorbed in cellulose membranes. The letters in the spectra designate the peaks of tenorite (T), cuprite (C), atacamite (A), nantokite (N), and malachite (M), and posnjakite (P), while asterisks denote the unknown peaks.

To understand components in the electrochemical corrosion, XRD analysis was performed (Figure 4). The peaks of copper were observed at 2θ values of 43.3° and 50.4° corresponding to (111) and (200) planes of Cu. Taking the composition of the corrosion solvent into account, the obtained spectra were compared to those of copper corrosions including oxide, carbonate, sulphate, and chloride. The absence of azurite (Cu_3_(OH)_2_(CO_3_)), brochantite (Cu_4_SO_4_(OH)_6_), antlerite (Cu_3_SO_4_(OH)_4_), and melanothallite (Cu_2_OCl_2_) was confirmed, while tenorite (CuO), cuprite (Cu_2_O), posnjakite (Cu_4_SO_4_(OH)_6_·H_2_O), atacamite (Cu_2_Cl(OH)_3_), nantokite (CuCl), and malachite (Cu_2_(OH)_2_CO_3_) were present. The spectra of these six components present in the corrosion are reported in Supplementary Material Figure S3. In the reference spectra, crystallographic planes of peaks, especially those visible in Figure 4, are also described. In the XRD pattern of the sample cleaned with DES70, some peaks (marked with asterisks), which cannot be assigned to any of the abovementioned compounds, were observed. The peaks related to the corrosion components were still noticeable when DES90 was used as a cleaning solvent. When DES80 was used, only the peaks related Cu(I)-based corrosions, such as cuprite and nantokite, were observed. This demonstrates the successful removal of Cu(II)-based corrosion products.

## 4. Discussion

It should be noted that DES is the mixture of two components which interact with each other not chemically but physically. The physical interactions can be disrupted by the addition of water. In the case of the DES based on ChCl and urea, the nanostructure of the DES is retained even at a remarkably high level of water content (ca. 42 wt% H_2_O) [14]. Above this concentration of water, however, the aqueous solution of DES is better described as a mixture of aqueous solutions of ChCl and Urea. Similarly, DES10, DES30, and DES50 should be considered as aqueous solutions dissolving ChCl and AA, separately, due to their large amount of water, i.e., the interactions between ChCl and AA are expected to be broken. In contrast to these, DES70, DES80, and DES90 can be classified as aqueous solutions of the DES.

For the sake of quick screening of the DES aq. solutions in terms of their cleaning ability of copper, dissolution tests of patina powder were carried out (Table 1). In addition, we assessed the solubility of calcium carbonate in the DES aq. because copper artifacts are often attached to limestone or marble bases, which contain calcium carbonate as a main component. Accordingly, the solutions with high patina solubility and low calcium carbonate solubility are favorable for the cleaning of copper artifacts. Complete dissolution of 1 wt% patina powder was achieved, except in DES10. The dissolution speed was fast in DES30, DES50, and DES70. The dissolution was expected to occur through the following reaction between patina and AA (malachite is taken as an example): Cu_2_(OH)_2_CO_3_ + 2C_6_H_8_O_6_ → 2CuC_6_H_6_O_6_ + CO_2_ + 3H_2_O. However, the solubilized state was not stable, and precipitations were formed in DES10, DES30, and DES50. Dissolution speed of patina powders in DES80 and DES90 was not fast, but the dissolution state was stable. In addition, these solutions exhibited a limited solubility of calcium carbonate suggesting that they were favorable for the removal of copper corrosion products from artifacts. In the solutions DES30, DES50, and DES70, the interactions between ChCl and AA were expected to be broken due to the presence of a large amount of water. Because of this, AA was free and readily available for the aforementioned reaction. Therefore, the dissolutions of both patina and calcium carbonate were fast. In contrast to this, in the highly concentrated solutions of DES80 and DES90, the interaction between AA and ChCl was present and controlled the dissolution, especially for calcium carbonates. From these facts, it was suggested that ChCl served a function to control the reactivity of AA through the formation of the deep eutectic mixture. Since DES70, DES80, and DES90 showed a good solubility of patina powders without the formation of precipitation, among which DES80 and DES90 were less harmful for calcium carbonates, these solutions were used for the cleaning tests.

For the cleaning tests, electrochemically corroded copper sheets were used. As shown in Figure 4, the corroded samples were found to contain tenorite, cuprite, atacamite, nantokite, malachite, and posnjakite. Since the copper sheet was oxidized at 0.150 V (vs. Ag/AgCl) and then at 0.380 V (vs. Ag/AgCl), Cu(I)-based corrosions such as cuprite should be formed on the surface of metallic copper, and then Cu(II)-based corrosions such as malachite and atacamite are expected to be formed on the cuprite surface, which can be explained by the Pourbaix diagram of Cu-Cl-CO_3_ [25].

**Figure 5:**
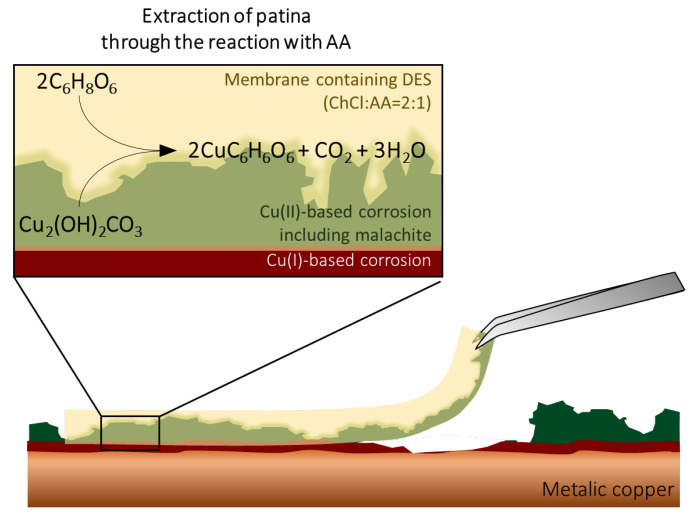
Schematic illustration of corrosion removal process of DES integrated with cellulose-based membranes.

Corrosion removal was carried out using cotton swabs and cellulose membranes containing selected DES aq. When the cleaning test was carried out simply by absorbing the DES aq. in the swabs, a better cleaning result in terms of the speed was obtained with DES70 compared to DES80 and DES90. This is because DES80 and DES90 possess a higher viscosity. Figure 5 shows the schematic illustration of the corrosion removal process by using a cellulose membrane containing the DES aq. When the membrane is placed on the corrosion, the reaction between the corrosion products (such as malachite) and DES (more specifically AA) is expected to occur as discussed above for the dissolution test. Through the reaction, corrosion is extracted into the membrane and taken off by removing the membrane. In the case of the cleaning using membranes, a shorter time was required in order to remove the malachite layer by using DES70. However, DES70 bled out from the membrane and changed the color of patina, which did not contact with the membrane. Consequently, area selectivity of the corrosion removal was not good. For the cleaning with cellulose membranes, well-balanced results in terms of speed and area selectivity were obtained when DES80 was used as the cleaning solvent. Additionally, from the XRD analysis, the removal of Cu(II)-based corrosions by DES80 was confirmed without the side effect observed when DES70 was used.

In this study, two different techniques were used for corrosion removal from copper. The swab was useful for a quick cleaning (five cycles of cleaning took less than 1 h). In addition, since the swab cleaning method needs to be carried out by human hands, the level of cleaning can be controlled by conservators. However, it consumes a larger amount of cleaning solution (e.g., 60 mg in five cycles), while the amount of DES aq. absorbed in the cellulose membrane with 1 cm diameter was at most 33 mg. In addition, it is necessary to apply a slight force to wipe out the patinas, which potentially deteriorate fine structures in the case of fragile ancient artifacts. The use of the membrane enables the retention of the DES aq. in the selected area by keeping the solution in the polymer matrices. The cleaning area can be defined by cutting the membranes into varying shapes and sizes depending on the requirements. The membrane allows the cleaning of hard-to-reach places because the cleaning can be carried out by solely applying the membrane to the object. However, this technique requires time to synthesize the membrane. For the conservation of copper, these two means must be properly used depending on the requirements and circumstances.

## 5. Conclusions

Greener and safer materials for the corrosion removal from copper, merely containing bio-derived materials, were developed in this study. As a standard material for cleaning, electrochemically corroded copper containing malachite and cuprite was prepared. DES composed of ChCl and AA was used as a cleaning agent. The aqueous solutions with 80 and 90 wt% DES (DES80 and DES90) were found to be effective for the complete dissolution of 1 wt% patina but not CaCO_3_. These solutions integrated into a cellulose membrane were applied on the surface of electrochemically corroded copper. After a few hours, the green corrosion layer, including malachite, was successfully removed by removing the membrane. When the cleaning was continued for 1 day, a surface of metallic copper became uncovered. Quick and high area-selective cleaning was accomplished by using the cellulose-based membrane with DES80. By XRD analysis of the corroded copper after cleaning, the absence of Cu(II)-based corrosions was confirmed. Therefore, the developed system represents an effective, green, and safe cleaning means for metallic materials, paving the way for the use of DES in metal preservation.

## Figures and Tables

**Table 1:** Time required for the complete dissolution of patina and calcium carbonate in the series of DES aq.

Concentration of DES (wt%)	Patina Powders	Calcium Carbonate
10	No complete dissolution ^1^	No complete dissolution
30	<1 h ^2^	<1 h
50	<1 h ^2^	<1 h
70	<1 h	<1 h
80	1 day	No complete dissolution
90	4 days	No complete dissolution

^1^ Blue precipitation was formed before the complete dissolution; ^2^ Greenish-blue precipitation was formed after 1 day and 4 days of the complete dissolution in DES30 and DES50, respectively.

## Data Availability

The data presented in this study are openly available in the digital repository of scientific publications of the University of Évora (https://dspace.uevora.pt/rdpc/), http://hdl.handle.net/10174/28982 (accessed on 17 May 2022).

## Supplementary Materials

The following supporting information can be downloaded at: https://www.mdpi.com/article/10.3390/polym14112284/s1, Figure S1: Photos taken during the course of dissolution test of patina powders; Figure S2: Photos taken during the course of dissolution test of calcium carbonate; Figure S3: XRD spectra of reference samples.
