# Supplementary material for: Removal of Copper Corrosion Products by Using Green Deep Eutectic Solvent and Bio-Derivative Cellulose Membrane
Source: Polymers (Basel). 2022 Jun 4;14(11):2284. doi: 10.3390/polym14112284 (PMC9182783; doi:10.3390/polym14112284)
Supplement: Supplementary file 1 [file polymers-14-02284-s001.zip › polymers-1753043-supplementary.pdf]

Removal of Copper Corrosion Products by Using Green Deep Eutectic Solvent and Bio-Derivative Cellulose Membrane

Akiko Tsurumaki[1,2,*], Cristina Chiarucci[1], Shraddha Khaire[1], Chiara Dal Bosco[1], Alessandra Gentili[1,2], and Maria Assunta Navarra[1,2,*]

[1] Department of Chemistry, Sapienza University of Rome, Piazzale Aldo Moro 5, Rome 00185, Italy;

[2] Centro di Ricerca Hydro-Eco, Department of Basic and Applied Sciences for Engineering (SBAI), Sapienza University of Rome, Via Antonio Scarpa 16, 00161 Roma, Italy.

* Correspondence: akiko.tsurumaki@uniroma1.it (A.T.); mariassunta.navarra@uniroma1.it (M.A.N.); Tel.: +39-06-4991-3664 (A.T.); +39-06-4991-3658 (M.A.N.)

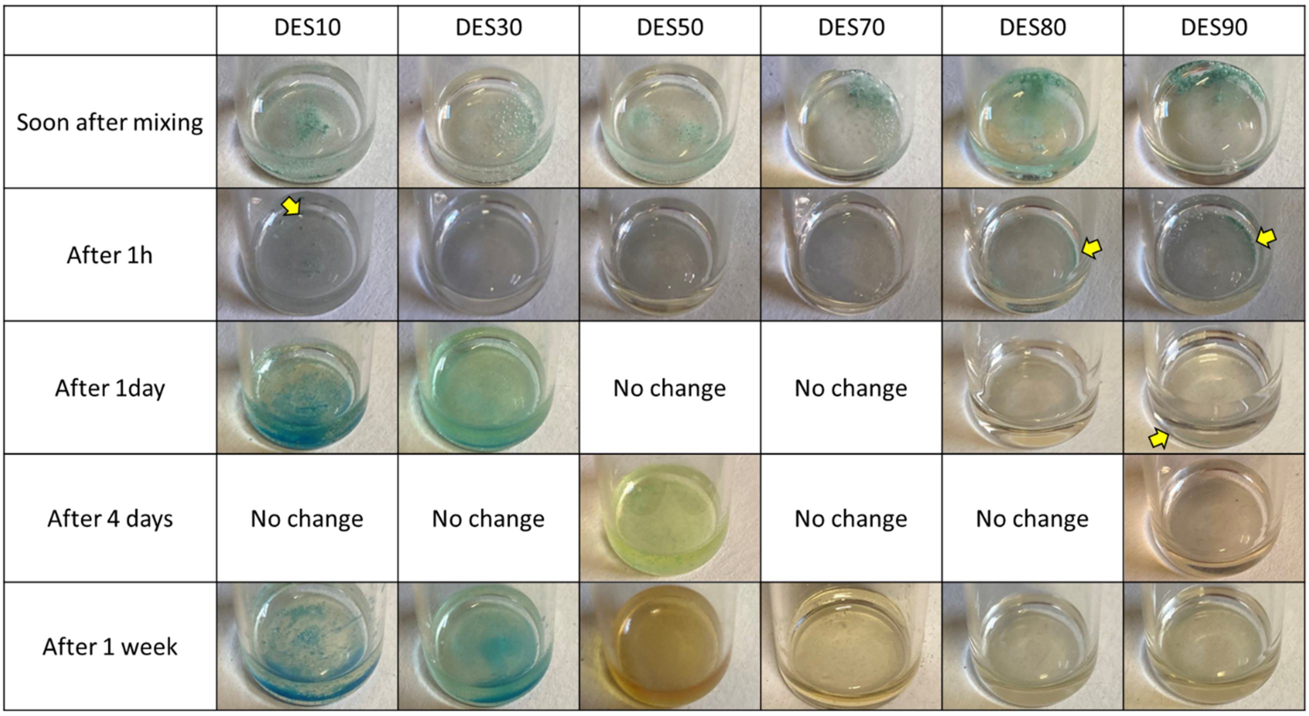

Figure S1. Photos taken during the course of dissolution test of patina powders. DES10: Formation of blue precipitation after partial dissolution of patina. DES30: Formation of blue precipitation after complete dissolution. DES50: Formation of greenish blue precipitation after complete dissolution, which was then turned into brown.DES70: Color change after complete dissolution but no formation of precipitation. DES80 & 90: Slow but complete dissolution. No change in the solution color after 1 week.

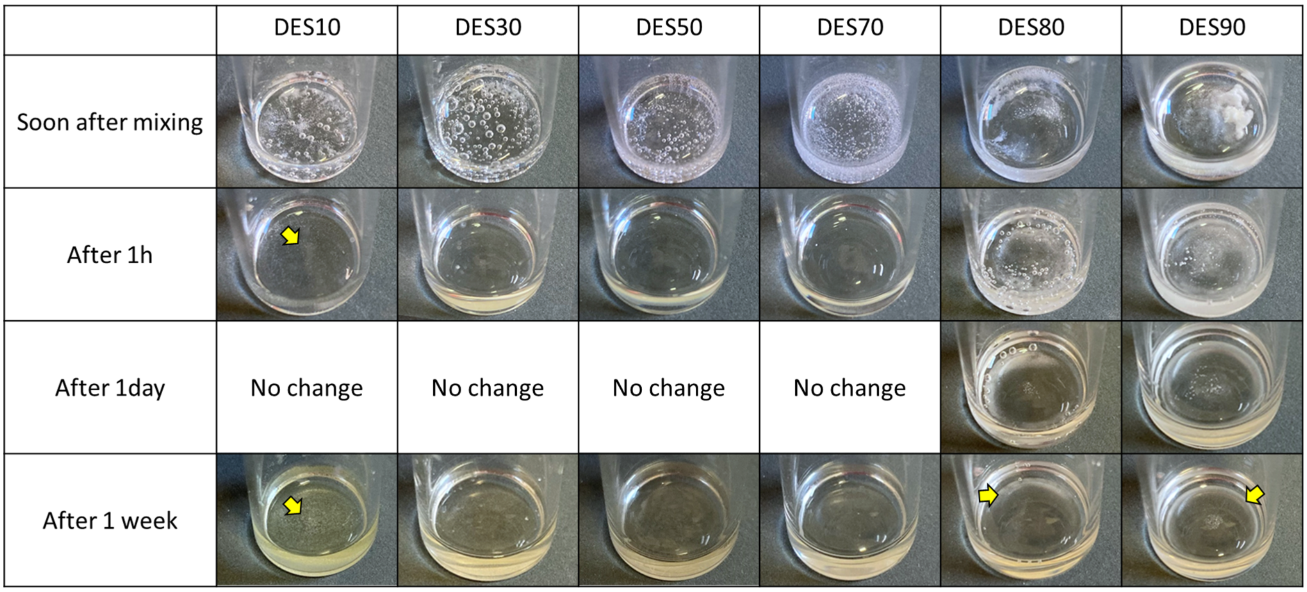

Figure S2. Photos taken during the course of dissolution test of calcium carbonate. DES10: Trace amount of $CaCO_3$ remained in the bottom of vial. DES30, 50 & 70: Fast and complete dissolution. DES80 & 90: Incomplete dissolution even after 1 week.

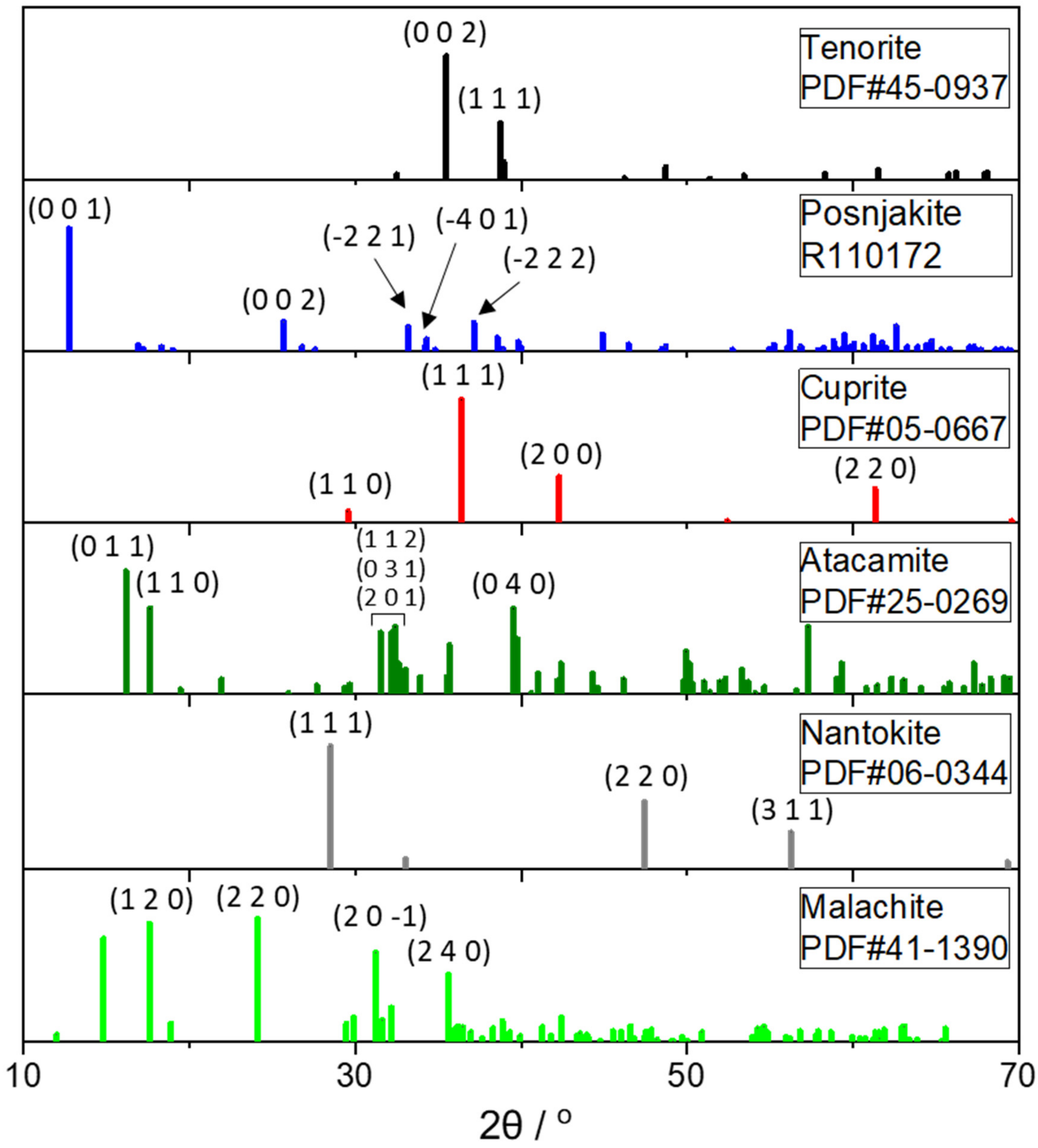

Figure S3. Reference spectra used for the assignment of Figure 4 (tenorite PDF#45-0937, cuprite PDF#05-0667, atacamite PDF#25-0269, nantokite PDF#06-0344, and malachite PDF#41-1390 taken from the International Centre for Diffraction Data (ICDD), as well as posnjakite R110172 taken from the RRUFF database).
